# A Case Series of Four Dogs Presenting with Neurological Deficits Due to Suspected Nutritional Secondary Hyperparathyroidism after Being Fed an Exclusive Diet of Raw Meat

**DOI:** 10.3390/ani14121783

**Published:** 2024-06-13

**Authors:** Lina Nowak, Suzanne van Loon, Esther Hagen-Plantinga, Niklas Bergknut

**Affiliations:** 1 The IVC Evidensia Referral Hospital Helsingborg, 254 66 Helsingborg, Sweden; 2 The IVC Evidensia Referral Hospital Hart van Brabant, 5144 AM Waalwijk, The Netherlands; 3 Nutrissues BV, Pet Nutrition Consultancy, 7422 RC Deventer, The Netherlands

**Keywords:** canine, nutritional secondary hyperparathyroidism, osteopenia, tetraparesis

## Abstract

**Simple Summary:**

Nutritional secondary hyperparathyroidism in dogs arises from either a deficiency in vitamin D or an improper calcium-to-phosphorus ratio in their diet. This report covers four cases of large-breed puppies fed exclusively boneless, non-supplemented raw meat diets. These puppies exhibited severe pain and difficulty walking. Imaging studies, including radiographs and computed tomography scans, showed decreased bone density, and two of the puppies suffered pathological fractures, resulting in their euthanasia. The other two puppies recovered quickly after receiving pain relief and a balanced commercial diet. Nutritional secondary hyperparathyroidism is triggered by low levels of active vitamin D and calcium, causing elevated parathyroid hormone levels in the blood to correct mineral imbalances. This mechanism includes pulling calcium from the bones, making the bones weak and increasing fracture risk. Once uncommon due to balanced commercial dog foods, nutritional secondary hyperparathyroidism has reappeared with the trend of feeding raw meat diets. Timely diagnosis and treatment are vital for recovery, highlighting the necessity of balanced diets to prevent serious skeletal and neurological problems in developing puppies.

**Abstract:**

Nutritional secondary hyperparathyroidism (NSH) in dogs is a condition that develops in response to a vitamin D deficiency or an imbalanced calcium-to-phosphorus ratio in dog food. Puppies of large-breed dogs exclusively fed a non-supplemented, boneless raw meat diet are especially susceptible to developing NSH due to their elevated calcium requirement. Reports on NSH in companion animals have been sparse in the last decades due to dog owners having easy access to commercially balanced dog foods. However, with the rising popularity of meat-based raw feeding, this condition has re-emerged. In this case series, four large-breed puppies fed exclusively non-supplemented, boneless raw meat diets presented with complaints of acute onset of pain and paresis. Radiographs and/or computed tomography (CT) scans showed reduced radio density of the skeleton in all four puppies. Two of the dogs had pathological fractures, and these two puppies were euthanized. One was subjected to a post mortem examination, which revealed cortical bone resorption and hypertrophy of the parathyroid glands. The remaining two puppies rapidly improved after receiving pain medication and a commercial, balanced diet. This case series demonstrates a risk of young dogs developing severe neurological deficits when fed a non-supplemented, boneless raw meat diet.

## 1. Introduction

Nutritional secondary hyperparathyroidism (NSH) is a condition in which the parathyroid gland produces supranormal amounts of parathyroid hormone (PTH) due to vitamin D deficiency or imbalanced calcium and phosphorus concentrations in the diet [1,2,3].

In response to deficiencies in active vitamin D, 1,25-dihydroxy vitamin D (1,25(OH) 2D), and/or extracellular calcium ion concentrations in the bloodstream, the parathyroid gland upregulates the production of PTH to maintain the appropriate levels of these minerals. Grossly simplified, the parathyroid glands increase the secretion of PTH to (1) increase the dietary absorption of calcium and phosphorus; (2) reduce urinary calcium excretion, inhibit phosphorus reabsorption, stimulate the production of (1,25(OH) 2D); and (3) increase the osteoclastic calcium mobilization to increase the efflux of calcium and phosphorus [1,2,3,4].

In NSH, the intestinal absorption of calcium is decreased due to low concentrations of 1,25(OH) 2D. A poor dietary intake of vitamin D exacerbates the decreased absorption of calcium from the gut. The breakdown of bone tissue is increased, leading to weakened bones and the risk of fractures. The metabolism of both calcium and 1,25(OH) 2D is disrupted, eventually leading to low serum calcium concentrations and low concentrations of 1,25(OH) 2D [2,3,4,5,6].

NSH is well described in dogs [6,7,8,9,10,11,12,13,14,15,16,17,18,19,20,21]. Puppies of large-breed dogs are more susceptible to developing NSH due to an increased calcium requirement [5,7,8,9,10]. An imbalanced diet has been described to cause osteoclastic bone resorption with cortical thinning, pathological fractures, and fibrous tissue replacement of bone [2,3,4,5,6,7,8,9,10]. Neurological symptoms such as pain and paresis have been reported [14,17,21]. NSH can be prevented and treated with proper nutrition and supplementation. A varied and balanced diet that provides adequate concentrations of calcium, phosphorus, and vitamin D is essential. In cases where dietary adjustments are not enough, supplemental calcium and vitamin D may be necessary. If diagnosed early, the prognosis for the patient is good [8,14,18]. Historically, NSH used to be more prevalent in companion animals, but with the increased availability of balanced, commercial pet food, NSH became a rare disorder. The rising popularity of raw meat diets has led to an increase in reported cases of NSH [3,6,8,11,15,18]. Early diagnosis is critical for the disease outcome and recovery of the patient. It is thus important to report suspected NSH cases and the way they present themselves to create renewed awareness among veterinarians.

## 2. Case Descriptions

Four puppies fed exclusively with non-supplemented, boneless raw meat diets presented with complaints of acute onset of pain and paresis.

The first puppy, dog A, was a 10-week-old intact male Golden Retriever with a weight of 3.3 kg. He had a 10-day history of progressive paresis, lethargy, hyporexia, and pain. He presented with non-ambulatory tetraparesis, reduced postural reactions, reduced muscle tone, weak spinal reflexes, and mild pain on palpation of the cervical and thoracic spinal cord. Based on these signs, the neuroanatomical localization was suspected to be the peripheral nervous system. The puppy was fed exclusively with a non-supplemented, boneless raw meat diet. 

The second puppy, dog B, was a 10-week-old intact male Kavkazskaja Ovtjarka weighing 10.4 kg. He had a history of twenty-four-hour signs of progressive pain, lethargy, and paresis. He presented with non-ambulatory paraparesis, reduced spinal reflexes in the hindlegs, and pain on palpation of the lumbar and sacral areas. The neuroanatomical localization was suspected to be the lumbar spinal segment. The puppy was fed a diet consisting of mainly raw meat, without bones or supplements. 

The third puppy, dog C, was a 12-week-old intact male Old English Bulldog and weighed 6.4 kg. He had a 2-day history of anorexia, pain, and lethargy. The puppy presented with pain and ambulatory tetraparesis. Spinal reflexes were normal. The neuroanatomical localization was suspected to be the cervical spinal cord, although a complete neurological assessment was challenging. The dog was exclusively fed raw meat. 

The fourth puppy, dog D, was a nine-week-old intact male Louisiana Catahoula Leopard Dog. He weighed 8.3 kg and presented with acute non-ambulatory paraparesis and pain after suspicion of acute trauma. He was painful on palpation over the pelvic and lumbar areas. The neuroanatomical localization was suspected to be the lumbar spinal segment. The puppy was fed a boneless raw meat diet.

In case A, the neuroanatomical localization was identified as the peripheral nervous system. The primary differential diagnoses included polyneuropathy resulting from infectious, immune-mediated, or metabolic etiologies or immune-mediated polyradiculopathy. Junctionopathy, such as in Myasthenia Gravis Syndrome, was also possible but deemed less likely due to the gradual, consistent, and linear progression of signs and the presence of pain in the pup. 

In cases B–D, the neuroanatomical localization was determined to be the spinal cord. The main differential diagnoses included suspected fractures or luxations, despite the absence of reported trauma. Other potential diagnoses, although less likely, included discospondylitis and polyarthritis. Additionally, nutritional vitamin D deficiency, nutritional secondary hyperparathyroidism (NSH), and autoimmune meningomyelitis could not be excluded.

## 3. Diagnostic Work-Up, Therapeutic Intervention, and Outcome

**Figure 1:**
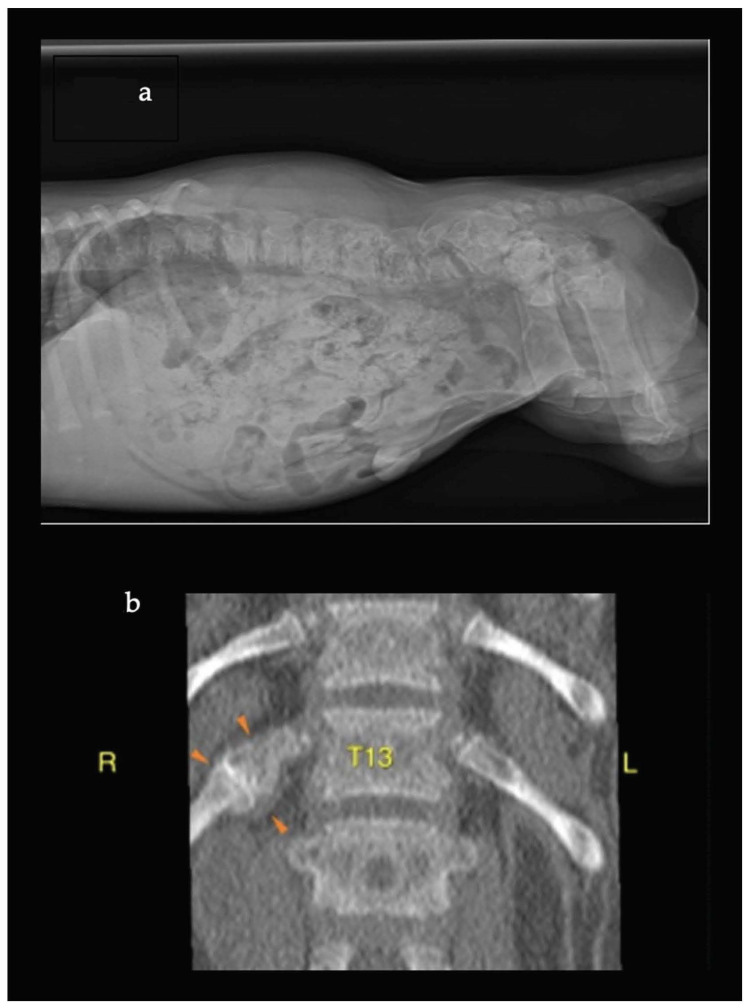
Radiographic images of dog A and dog D. Radiographic image (latero-lateral view) of the lumbosacral area of dog A (**a**) shows a suspected general low radiodensity of the bones. Computed tomography image (dorsal view) of the T12–L2 area of dog D (**b**) reveals generalized osteopenia and an old fracture of the 13th rib on the right side (indicated by amber arrowheads).

In dog A (Table S1), the diagnostic work-up was based on a suspected disease of the peripheral nervous system. Hematological testing with a complete blood cell count (CBC) showed leukocytosis, and biochemistry analysis revealed increased concentration of C-reactive protein (CRP). Creatine kinase (CK) and total calcium and phosphorus concentrations were all normal. A urine analysis showed no clinically significant abnormalities. Radiographs of the thorax, abdomen, cervical spine, and long bones were obtained. Initially, these radiographs were interpreted by the veterinary surgeon and reported as normal, with a suspicion of mildly radiolucent bones (Figure 1a). This suspicion was later confirmed by a board-certified diagnostic imaging veterinarian. A cisternal cerebrospinal fluid (CSF) sample showed a mild mixed pleocytosis (Table 1). The owners elected euthanasia, and the dog was referred for a post mortem investigation. The pathology report revealed a severe loss of bone mineralization and mildly diffuse and hypertrophic parathyroid glands. There were no pathological changes in the nervous system to explain the progressive paresis. There was a myeloid proliferation of the bone marrow, and the suspected clinical diagnosis was NSH.

**Figure 2:**
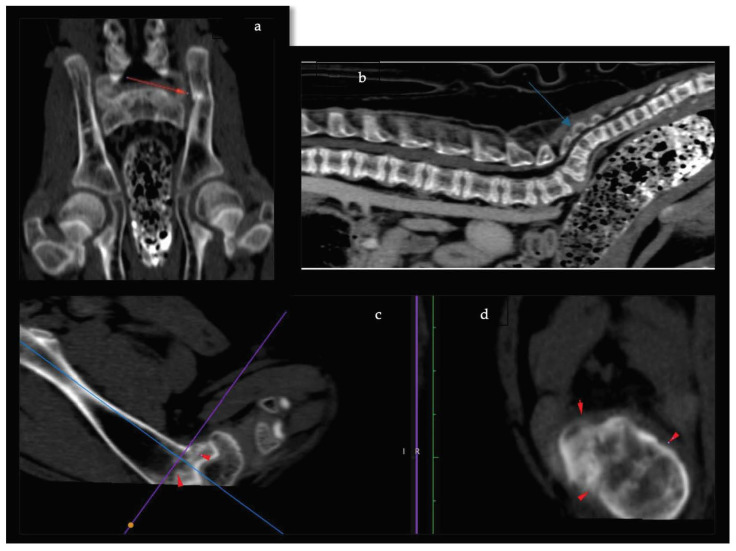
Computed tomography images of dog B. Computed tomography images, dorsal view (**a**) and sagittal view (**b**), of the pelvis and sacrum of dog B show a folding fracture of the left ilium (red arrow, (**a**)) and a curved sacrum (blue arrow, (**b**)). Note the decreased density/attenuation of the vertebrae. Images of the left distal femur in sagittal view (**c**) and dorsal view (**d**) indicate suspected abnormal femoral metaphysis with a possible folding fracture at this level (red arrowheads).

In dog B (Table S2), the diagnostic work-up with hematology testing showed mild anemia and thrombocytosis on the CBC, increased CRP, and slightly increased phosphorus on the biochemistry. Blood calcium was within normal limits (Table 1). Diagnostic imaging was focused on the lumbosacral spine and the sacrum. Computed tomography (CT (Philips Big Bore, 16 Helical Slices)), showed general bone radiolucency, a folding fracture of the left ilium, a possible folding fracture on the left distal femur, and a curved sacrum (Figure 2a–d). The owners elected euthanasia due to the severity of the clinical signs and declined a post mortem investigation.

In dog C (Table S3), the CBC and the biochemistry testing were within normal limits (Table 1). Diagnostic imaging with radiographs of the spinal cord and abdomen revealed general bone radiolucency. The puppy was treated with gabapentin 8 mg/kg three times daily and meloxicam 0.1 mg/kg once daily. The diet was altered to a balanced commercial dog food. The puppy recovered clinically within two days. There was no long-term follow-up.

Dog D (Table S4) had normal results on biochemistry, apart from an increased concentration of PTH. Serum calcium and ionized calcium were normal; phosphorus was slightly increased (Table 1). For further evaluation of the acute neurological abnormalities, CT (Philips Gantry Brilliant Big Bore, 16 Helical Slices, Philips Medical Systems, Cleveland, Ohio 44143 USA) of the thoracolumbar and lumbosacral spine was performed. The CT showed generalized bone radiolucency and revealed an old fracture of a rib (Figure 1b). The puppy received gabapentin 10 mg/kg three times daily, meloxicam 0.1 mg/kg once daily, and dietary management. The diet was altered to a balanced commercial dog food, suited for rapidly growing puppies of large breeds. Follow-up showed a swift recovery (within days) and complete resolution of clinical signs. A follow-up examination three months later showed that the dog was still well without any medication. 

In conclusion, the four puppies were fed exclusively a non-supplemented, boneless raw meat diet and presented with complaints of acute onset of pain and paresis. Radiographs and/or CT showed reduced radio density of the skeleton in all four puppies. Two of the dogs had pathological fractures, one had a fractured rib, and the other had folding fractures of the pelvis and abnormal curvature of the sacrum.

Two puppies were euthanized. One was subjected to a post mortem examination, which revealed diffuse, severe cortical bone resorption and hypertrophy of the parathyroid glands. The remaining two puppies rapidly improved after receiving pain medication and a commercial, balanced diet.

## 4. Discussion

In this case series, four puppies of large breeds, all fed a non-supplemented, bone-free diet, presented with similar symptoms of pain and paresis. The suspected neuroanatomical localization was the peripheral nervous system in dog A and the spinal cord in dogs B–D. In previous reports on suspected NSH in dogs, the main neurological signs have been pain and/or paresis [14,17,21]. The neurological signs of NSH are described to be secondary to vertebral body fractures or possibly to altered concentrations of circulating blood calcium [2,16,17,18,22]. We assessed the neuroanatomical localizations based on the distribution of pain, the presence of paresis, and reduced spinal reflexes. However, the exact etiology remained unclear. The pain could have been due to fractures impinging on peripheral nerves or the spinal cord. Additionally, pain may have caused the dog to be unwilling to ambulate.

Imbalanced blood calcium concentrations can affect nerve function. Low calcium levels can lead to increased nerve excitability, causing muscle twitching or spasms. Conversely, high calcium concentrations can depress nerve activity, potentially leading to weakness or paresis in severe cases [2]. Circulating calcium refers to the total amount of calcium present in the bloodstream, including both ionized and protein-bound forms. Ionized calcium, on the other hand, represents the biologically active form of calcium that is readily available for use by nerves and muscles [2]. In this condition, there might be a discrepancy between circulating calcium concentrations and ionized calcium concentrations due to inadequate dietary calcium intake or imbalances in vitamin D metabolism, leading to increased PTH secretion and subsequent bone resorption. This can result in low ionized calcium concentrations, despite normal or even increased circulating calcium concentrations, impacting nerve and muscle function [2,23].

In these cases, two of the dogs (dog B and dog D) were found to have fractures. CT scans were performed on these dogs, offering superior contrast resolution and cross-sectional imaging, which allowed detailed evaluation of bone integrity and radiolucent lesions. This method provides a three-dimensional assessment, enabling a more comprehensive analysis of bone structure compared to traditional radiography [24,25].

None of the puppies had abnormal blood calcium concentrations. Ionized calcium was evaluated in one case and was found to be within normal values. Although less likely, it remains a possibility that imbalanced blood calcium contributed to the described paresis.

In humans, the neurological symptoms associated with NSH include easy fatigability, weakness, muscle atrophy, and pain, particularly in the proximal muscles, while reflexes are preserved in most cases. Neuromuscular symptoms have also been reported [22,26].

As described above, it is plausible that dogs with NSH could present with signs of pain and paresis, where neuroanatomical localization could be suspected to involve either upper motor neuron disease (dogs B–D) or lower motor neuron disease (dog A).

Radiography, despite being two-dimensional and having less contrast resolution compared to CT, remains a valuable diagnostic tool due to its accessibility and utility in initial assessments. The radiographic interpretation, recognizing patterns indicative of bone radiolucency, raised differentials including renal secondary hyperparathyroidism, osteogenesis imperfecta (OI), or NSH. Renal causes were ruled out based on normal kidney values in blood work-ups and the absence of characteristic radiographic findings, such as subperiosteal bone resorption or soft tissue calcifications [1,2,27]. Osteogenesis imperfecta is a genetic inherited disease characterized by defective collagen, resulting in brittle bones and fragile teeth. Affected puppies may experience bone fractures following minor trauma. While less common than NSH, OI must be considered if serum concentrations of ionized calcium, phosphorus, vitamin D, and PTH are within reference ranges [28].

Considering the presented cases, the likelihood of OI diminished with severe loss of bone mineralization and mildly enlarged parathyroid glands observed post mortem in dog A, and skeletal radiolucency with spontaneous recovery after a dietary change in dogs C and D. However, in dog B, where euthanasia occurred without a dietary change or post mortem examination, OI remained a possible diagnosis [28]. 

NSH is a condition stemming from chronic dietary deficiencies in vitamin D or calcium or an imbalance in the calcium-to-phosphorus ratio in the bloodstream. In the cases described, the puppies were fed non-supplemented, bone-free, all-meat diets. Such raw all-meat diets often lack vitamin D, are deficient in calcium, contain excessive phosphorus, and exhibit an inappropriate calcium-to-phosphorus ratio [19,29]. Feeding a diet deficient in vitamin D and calcium, with an excess of phosphorus, triggers hypocalcemia, prompting a compensatory response from the parathyroid gland. Growing large-breed puppies, with their high calcium demand and limited calcium reserves, are particularly susceptible [3,7,8,9,10,20]. Passive calcium absorption occurs from six weeks to six months of age, with intestinal absorption directly proportional to dietary calcium intake, accounting for up to 70% of calcium uptake [3,7,20]. After six months, as hormonal and intestinal regulation mature, active calcium absorption becomes predominant, contributing to 90% of calcium uptake. Thus, young puppies rely on adequate dietary calcium intake for proper calcium homeostasis. 

The primary limitation of this case series lies in the presumptive diagnosis of all the puppies. Because only one puppy underwent a post mortem examination, PTH was only measured and found to be increased in one instance, leaving some questions unanswered.

Beyond the nutritional causes of vitamin D deficiency, genetic familial factors may also contribute. For example, a recent publication by Rohdin et al. (2023) identifies abnormalities in bone formation in Pugs as linked to a mutation in CYP27B1, a gene involved in genetic disorders affecting vitamin D metabolism [5,30].

In these cases, the diagnoses were established based on a combination of clinical signs, radiographic/CT features, post mortem findings, and the exclusive meat-only diet of the puppies. Moreover, the two surviving puppies fully recovered on a balanced diet.

Another limitation of this case series is the absence of a detailed description and nutritional analysis of the puppies’ diets. However, all cases shared the commonality of being fed a non-supplemented, boneless, all-meat diet as their sole nutrition. A study by Krook and Whalen demonstrated that both puppies and tiger cubs exclusively fed boneless meat developed severe bone pain, marked osteopenia, and spontaneous fractures [6]. Nutritional analysis in that study revealed severe calcium deficiency, excess phosphorus, and a highly skewed calcium-to-phosphorus ratio of 1:25. It is thus highly likely that similar nutritional deficits were present in the puppy cases discussed here.

NSH was once more prevalent in dogs; however, the availability of numerous balanced commercial pet foods led to a decline in such cases [6,8,11]. The recent surge in the popularity of meat-only diets has brought this issue back to the forefront. If diagnosed early in the disease’s progression, the prognosis for complete recovery is favorable. Transitioning to a balanced diet, along with potential calcium supplementation, typically results in normalization of skeletal mineralization within 4–8 weeks [8,11,14].

In dogs C and D, the administration of pain medication and the transition to a commercial and balanced diet yielded a rapid recovery. We, therefore, did not supply any extra calcium and the puppies continued to improve and recover on this diet.

This case series underscores the relevance of considering suspected NSH as a potential differential diagnosis in growing puppies. It highlights the significant risk of young dogs developing severe neurological deficits, potentially attributable to NSH, when maintained on an exclusively non-supplemented, bone-free, raw meat diet. It is crucial to recognize that raw meat diets can vary substantially based on ingredient selection and their nutritional composition. These diets may include boneless alternatives, bone-enriched varieties, meat-exclusive options, or those incorporating modest amounts of plant-based ingredients. Furthermore, they may be either non-supplemented or enriched with added vitamins and minerals. Given the extensive range of commercial raw-meat-based offerings available to pet owners, veterinary professionals must determine the precise nature of the diet when owners opt for a raw-meat-based regimen for their pets. While some options may be nutritionally balanced through the inclusion of essential vitamins and minerals, a significant portion of raw meats provided to pets may lack one or more crucial nutrients [29,31,32]. A thorough nutritional history is essential to gather essential nutritional information and conduct a comprehensive assessment of the risk for nutritional imbalances.

## 5. Conclusions

Four puppies, fed exclusively a non-supplemented, boneless raw meat diet, presented with acute pain and paresis. Radiographs and/or CT scans showed reduced skeletal radiodensity in all four puppies. Two had pathological fractures: one with a fractured rib and the other with pelvic folding fractures and sacral curvature abnormalities. Two puppies were euthanized, with one post mortem examination revealing severe cortical bone resorption and parathyroid gland hypertrophy. The remaining two puppies rapidly improved with pain medication and a balanced commercial diet.

This case series highlights the risk of young dogs experiencing severe neurological deficits, such as sudden onset of pain and paresis, likely attributed to suspected nutritional secondary hyperparathyroidism (NSH) when fed a non-supplemented, boneless raw meat diet. Hence, ensuring a balanced diet is essential for all dogs, especially growing puppies. 

## Figures and Tables

**Table 1:** Schematic overview over relevant diagnostics in the four puppies, A–D.

Dog	Ca (mmol/L)	P (mmol/L)	CRP (mg/L)	PTH (mmol/L)	Diagnostic Imaging	CSF (Cells/Microliter)	Pathology	Outcome
**A**	2.4 (1.95–3.15)	1.87 (1.65–3.36)	58.3 (0–10)	-	X-ray	11	Yes	Euthanized
**B**	2.5 (2.0–2.8)	2.82 (0.8–2.0)	80 (0–30)	-	CT	-	No	Euthanized
**C**	2.98 (1.95–3.15)	2.31 (1.65–3.36)	9.6 (0–10)	-	X-ray	-	No	Full recovery
**D**	2.4 (2.0–2.8)	2.7 (0.9–1.7)	-	406 (20–65)	CT	-	No	Full recovery

Abbreviations: Ca = calcium, P = phosphorus, CRP = C-reactive protein, PTH = parathyroid hormone, X-ray = radiography, CT = computed tomography, CSF = cerebrospinal fluid.

## Data Availability

The original contributions presented in the study are included in the article/Supplementary Material, further inquiries can be directed to the corresponding author.

## Supplementary Materials

The following supporting information can be downloaded at: https://www.mdpi.com/article/10.3390/ani14121783/s1, Table S1: Hematology, biochemistry, and urinary test, dog A. Table S2: Hematology and biochemistry, dog B. Table S3: Hematology and biochemistry, dog C. Table S4: Biochemistry, dog D.
