# Supplementary material for: A Case Series of Four Dogs Presenting with Neurological Deficits Due to Suspected Nutritional Secondary Hyperparathyroidism after Being Fed an Exclusive Diet of Raw Meat
Source: Animals (Basel). 2024 Jun 13;14(12):1783. doi: 10.3390/ani14121783 (PMC11200917; doi:10.3390/ani14121783)
Supplement: Supplementary file 1 [file animals-14-01783-s001.zip › animals-3043247-supplementary.pdf]

Supplementary Material:

Table S1. Hematology, biochemistry, and urinary test, dog A.

**BAND**	SUSPECT		
**%NEU**	SUSPECT 51.8	%	
**RETIC**		K/μL	(10.0 - 110.0)
**%RETIC**		%	
**PDW**		fL	(9.1 - 19.4)
**MCHC**	34.6	g/dL	(32.0 - 37.9)
**%BASO**	0.0	%	
**PCT**	0.18	%	(0.14 - 0.46)
**PLT**	SUSPECT 123.0	K/μL	(148.0 - 484.0)
**RDW**	19.9	%	(13.6 - 21.7)
**%EOS**	0.2	%	
**MCH**	20.9	pg	(21.2 - 25.9)
**HCT**	21.7	%	(37.3 - 61.7)
**LYM**	SUSPECT 36.98	x10^9/L	(1.05 - 5.1)
**NEU**	SUSPECT 67.84	x10^9/L	(2.95 - 11.64)
**MONO**	SUSPECT 25.73	x10^9/L	(0.16 - 1.12)
**MPV**	14.3	fL	(8.7 - 13.2)
**RETIC-HGB**		pg	(22.3 - 29.6)
**RBC**	3.59	x10^12/L	(5.65 - 8.87)
**MCV**	60.4	fL	(61.6 - 73.5)
**%LYM**	SUSPECT 28.3	%	
**BASO**	0.03	x10^9/L	(0.0 - 0.1)
**HGB**	7.5	g/dL	(13.1 - 20.5)
**%MONO**	SUSPECT 19.7	%	
**EOS**	0.25	x10^9/L	(0.06 - 1.23)
**WBC**	130.83	x10^9/L	(5.05 - 16.76)
**AMYL**	348.0	U/L	(300.0 - 1300.0)
**Osm Calc**	289.0	mmol/kg	
**CHOL**	3.92	mmol/L	(2.58 - 10.32)

**ALB/GLOB**	1.2		
**GGT**	4.0	U/L	(0.0 - 2.0)
**Cl**	111.0	mmol/L	(105.0 - 119.0)
**BUN/CREA**	67.0		
**K**	4.3	mmol/L	(3.5 - 5.5)
**PHOS**	1.87	mmol/L	(1.65 - 3.36)
**TT4**	21.0	nmol/L	(13.0 - 51.0)
**ALT**	42.0	U/L	(8.0 - 75.0)
**LIPA**	626.0	U/L	(100.0 - 1500.0)
**Na/K**	34.0		
**CREA**	12.0	µmol/L	(27.0 - 106.0)
**TP**	49.0	g/L	(48.0 - 72.0)
**GLU**	5.72	mmol/L	(4.28 - 8.34)
**CRP**	58.3	mg/L	(0.0 - 10.0)
**ALB**	27.0	g/L	(21.0 - 36.0)
**TBIL**	< 2.0	µmol/L	(0.0 - 14.0)
**ALKP**	255.0	U/L	(46.0 - 337.0)
**Na**	146.0	mmol/L	(145.0 - 157.0)
**GLOB**	22.0	g/L	(23.0 - 38.0)
**CK**	238.0	U/L	(99.0 - 436.0)
**CA**	2.4	mmol/L	(1.95 - 3.15)
**UREA**	3.3	mmol/L	(2.5 - 10.4)

**Urine pH**	**5.0**	
**UBG**		Normal
**Clear**		
**GLU**		Negative
**BLD**		1+
**Collec**		Spontaneous
**LEU**		Negative
**KET**		Negative
**PRO**		Trace
**Color**		Pale yellow
**BIL**		Negative
**SG**	1.018	

Table S2. Hematology and biochemistry, dog B

**HGB**	B-Hemoglobin, Hb	12.4	13.10 - 20.50	g/dL
**RBC**	B-Erytrocythalt (RBC)	5.50	5.65 - 8.87	x10^12/L
**WBC**	B-Leukocyter	16.48	5.05 - 16.76	x10^9/L
**1NEUTR**	-Totalantal neutrofiler	6.8*	3.00 - 11.50	x10(9)/L
**2TOTSTAV**	-Totalantal stavar	0.3	0.00 - 0.30	x10(9)/L
**3TOTEOS**	-Totalantal eosinofiler	0.7	0.00 - 1.25	x10(9)/L
**5BASO**	-Totalantal basofiler	0	0.00 - 0.00	x10(9)/L
**7TOTLYMF**	-Totalantal lymfocyter	6.6	1.00 - 4.80	x10(9)/L
**8TOTMONO**	-Totalantal monocyter	2.0	0.00 - 1.35	x10(9)/L
**9TOTOMOG**	-Totalantal omogna	0.2	0.00 - 0.00	x10(9)/L
**KÄRNF ERY**	-Kärnförande erytrocyter	0		
**RÖD MORF**	-Röd morfologi	normal		
**MCV**	MCV	68.0	61.60 - 73.50	fL
**MCH**	MCH	22.5	21.20 - 25.90	pg
**MCHC**	MCHC	33.2	32.00 - 37.90	g/dL
**HCT**	B-Hematokrit, HT	37.4	37.30 - 61.70	%
**RETIC**	B-Retikulocyter totalantal	34.1	10.00 - 110.00	K/μL
**TROMBOCYT**	B-Trombocyter	554	148.00 - 484.00	K/μL
**MPV**	Blodplättsvolym (MPV)	12.0	8.70 - 13.20	fL
**CRP HD**	S-CRP Hund LAB	80	0.00 - 30.00	mg/l
**KREA**	S-Kreatinin	35	65.00 - 105.00	μmol/l

Table S3. Hematology and biochemistry, dog C.

ProCyte_Dx

Instrument Notes: Anemie zonder reticulocytose – waarschijnlijk non-regeneratieve anemie; denk aan pre-regeneratieve anemie.

BASO	0.08	x10^9/L	(0.0 - 0.1)
RETIC-HGB	22.5	pg	(22.3 - 29.6)
MCH	23.4	pg	(21.2 - 25.9)
MCV	63.7	fL	(61.6 - 73.5)
RDW	17.8	%	(13.6 - 21.7)
HGB	10.3	g/dL	(13.1 - 20.5)
%LYM	24.6	%	
%BASO	0.7	%	
EOS	0.11	x10^9/L	(0.06 - 1.23)
RBC	4.41	x10^12/L	(5.65 - 8.87)
WBC	11.25	x10^9/L	(5.05 - 16.76)
PCT	0.48	%	(0.14 - 0.46)
NEU	7.22	x10^9/L	(2.95 - 11.64)
HCT	28.1	%	(37.3 - 61.7)
%RETIC	0.5	%	
%EOS	1.0	%	
PDW	10.9	fL	(9.1 - 19.4)
LYM	2.77	x10^9/L	(1.05 - 5.1)
MONO	1.07	x10^9/L	(0.16 - 1.12)
RETIC	19.8	K/µL	(10.0 - 110.0)
MPV	10.6	fL	(8.7 - 13.2)
MCHC	36.7	g/dL	(32.0 - 37.9)
%MONO	9.5	%	
PLT	451.0	K/µL	(148.0 - 484.0)
%NEU	64.2	%	

Catalyst_One

Instrument Notes: C-Reactief Proteïne (CRP) CRP-concentraties >30,0 mg/l duiden op een klinisch significante systemische ontsteking.

ALT	27.0	U/L	(8.0 - 75.0)
CHOL	9.21	mmol/L	(2.58 - 10.32)
AMYL	564.0	U/L	(300.0 - 1300.0)
LIPA	365.0	U/L	(100.0 - 1500.0)
TP	56.0	g/L	(48.0 - 72.0)
GLOB	27.0	g/L	(23.0 - 38.0)
ALB/GLOB	1.1		
CREA	52.0	µmol/L	(27.0 - 106.0)
BUN/CREA	18.0		
GGT	0.0	U/L	(0.0 - 2.0)
UREA	3.7	mmol/L	(2.5 - 10.4)
TBIL	4.0	µmol/L	(0.0 - 14.0)
GLU	6.62	mmol/L	(4.28 - 8.34)
CRP	9.6	mg/L	(0.0 - 10.0)
ALB	29.0	g/L	(21.0 - 36.0)
ALKP	187.0	U/L	(46.0 - 337.0)
CA	2.98	mmol/L	(1.95 - 3.15)
PHOS	2.31	mmol/L	(1.65 - 3.36)

ProCyte_Dx

Table S4. Biochemistry, dog D.

S-ALAT	0.30<br>0.30 - 1.30 µkat/l
S-Kreatinin	35<br>65.00 - 105.00 µmol/l

Parathormone intact - idexx	406<br>20.00 - 65.00 pg/ml
S-Fosfat	2.7 (0.9-1.7) mmol/l
S-Calcium	2.4 2.00 - 2.80 mmol/l
B-joniserat Calcium	1.20<br>1.12 - 1.42 mmol/l
